# Twinning in Italian Holstein Cows: A Retrospective Study on Risk Factors and Its Associations with Milk Production, Fertility, and Survival

**DOI:** 10.3390/vetsci12040353

**Published:** 2025-04-10

**Authors:** Marcello Guadagnini, Paola Amodeo, Agostino Bolli, Monica Probo

**Affiliations:** 1 Axiota Animal Health, 2809 East Harmony Road #190, Fort Collins, CO 80528, USA; marcello.guadagnini@multimin.eu; 2 Dairy Science Specialist, Via Carpaccio 3, 20133 Milan, Italy; paamodeo@gmail.com; 3 Alta Italia s.r.l., Via Mascherpa, 10 Paullo, 20067 Milan, Italy; agostino.bolli@altagenetics.com; 4 Department of Veterinary Medicine and Animal Sciences, Università degli Studi di Milano, Via dell’Università 6, 26900 Lodi, Italy

**Keywords:** twinning, Italian Holstein, risk factor, fertility, milk production, culling risk

## Abstract

Twinning in cattle is an increasingly common reproductive phenomenon that has significant implications for herd productivity and animal welfare. This retrospective study aimed to assess the occurrence rate of twinning (TW) in Italian Holstein multiparous cows and to identify the associated risk factors and consequences with respect to fertility, milk production, and cow survival. Data from 44,535 calvings across 61 Italian dairy farms from January 2019 to December 2020 were analyzed. The overall TW rate was found to be 3.79%, affected by factors such as parity (*p* < 0.0001), season of conception (*p* < 0.0001), previous lactation 305-day mature equivalent (*p* < 0.05), and prior days open (*p* < 0.0001). Cows experiencing TW had significantly shorter gestation lengths (*p* < 0.0001) compared to singletons and lower 305-day milk production estimates (11,094 kg for TW vs. 11,295 kg for singleton females and 11,318 kg for singleton males) (*p* < 0.0001). Moreover, TW was linked to a decreased pregnancy risk and an increased risk of culling (*p* < 0.0001) both in short- and long-term analysis. These findings underline the need for targeted management strategies to mitigate the adverse effects of twinning in dairy herds, especially where TW rates are notably high.

## Simple Summary

Twinning in dairy cattle is becoming more common and raises important concerns about productivity and animal well-being. This study retrospectively looked at data from over 44,000 births across 61 dairy farms in Italy to understand risk factors and effects of twinning. About 3.79% of the cows had twins. Several factors influenced this rate, including the cow’s age, the season when the cow became pregnant, and their previous milk production. Cows that gave birth to twins had shorter pregnancies and lower milk production compared to those that had just one calf. Additionally, cows that had twins were less likely to become pregnant again and faced a higher chance of being culled from the herd. These results highlight the need for better management practices in dairy farming, especially in herds where twinning is frequent, to reduce the negative impacts on both the cows and the overall productivity of the farm.

## 1. Introduction

Twinning in cattle, defined as the simultaneous delivery of two calves, represents a reproductive phenomenon that deviates from the typical monovular calving pattern observed in cows. Cattle are predisposed to single births due to their reproductive biology; however, the incidence of twinning has significantly increased over the past few decades [1], potentially due to advances in genetic selection, nutrition, and reproductive management practices [2,3,4,5]. The selection for higher milk yield has inadvertently selected for traits associated with increased ovulation rates, contributing to higher occurrences of multiple ovulations and, consequently, twinning [6]. Furthermore, contemporary breeding techniques utilize hormonal protocols that can inadvertently raise the likelihood of double ovulation; while some short protocols seem to reduce the double ovulation rate compared to spontaneous estrus or to longer protocols [7,8], other hormone combinations may increase the risk of double ovulation [9]. Despite the potential economic benefits associated with increased calf production for beef cattle industry, twinning in the dairy cow is often associated with well-known adverse outcomes, including higher rates of obstetric complications [10], reduced calf viability [11], and increased health and survival risks for the cow [2,12]. Advances in transrectal ultrasonography have facilitated the early identification and classification of twin pregnancies, leading to a more detailed economic assessment of twinning impacts [13]. Recent analyses estimate the economic detriment of twinning to be around USD 161, on average, per instance, with the best management strategy after diagnosis being manual embryo reduction in order to mitigate losses [14]. As such, understanding the underlying mechanisms that influence twinning rates, as well as their implications for animal welfare and herd productivity, is crucial for optimizing reproductive performance in dairy herds. Continued research into the physiological, genetic, and environmental factors associated with twinning will be essential to manage its prevalence effectively and mitigate its associated risks within the dairy industry.

In Italy, the most reared dairy cattle breed is the Italian Holstein, counting for more than 1,000,000 live animals and about 9000 breeders, with an average of 10,802 kg of milk produced per lactation/cow in 2023 [15]. However, to the best of our knowledge, specific data about twinning in Italian Holstein herds are missing, while numerous reports are present in the literature regarding the incidence, risk factors, and consequences of twinning in dairy cows all over the world [2,16,17,18,19,20,21,22]. Therefore, the aim of this retrospective study was to describe twinning in Italian Holstein cows, addressing potential risk factors and measuring possible associations between twin calvings and fertility, culling, and milk production.

## 2. Materials and Methods

This retrospective observational study involved the analysis of data collected from cows calving between January 2019 to December 2020. The original dataset is the same used for Guadagnini et al. [23], and statistical analysis was performed using JMP17 (SAS Institute Inc., Cary, NC, USA). Recruitment criteria for dairy herds were as follows: the use of the herd management program Dairy Comp 305^®^ version 5.0 (Valley Ag Software, Tulare, CA, USA) to record herd data for at least the 2 previous years, and enrollment in the Italian National Breeders Association for milk production. According to these criteria, and on the basis of their consistency over time in recording twinning (TW), a total of 61 Italian dairy farms were enrolled in the retrospective analysis. Selection criteria for the present study were as follows: being a multiparous Holstein cow with at least one calving event from January 2019 to December 2020, with a previous gestation length between 260 and 300 days, and a known calving outcome. Therefore, parity 1 cows were excluded. Cows with parity ≥3 were all considered as unique group (LACT = 3+). Twinning was identified by two variables: “Twinning (Yes vs. No)” and “Calf outcome (Female vs. Male vs. Twins)”. Information regarding previous lactation 305-day mature equivalent (305PME) and 305-day milk estimate (305ME) in the current lactation was available in the dataset, as well as gestation length, dry period length, and conception date.

Based on previous lactation conception dates, a variable named season of conception (SEACON) was created, and previous lactation days open were classified in 3 categories: <100 days, 100–200 days, and >200 days. Descriptive statistics regarding TW occurrence were performed for the overall dataset by farm and lactation group. Least square means (LSM) for gestion length were calculated for calf outcome using a linear mixed model with gestation length as independent variable, 305MEP was a covariate, calving month, calving year, calf outcome, parity group, and calf outcome by parity group interaction as fixed dependent variables. The farm variable was included as a random effect. Aiming to address the risk factors for TW, a multiple logistic regression model was created using twinning (Yes vs. No) as independent variable and farm, parity group, SEACON, previous days open category, 305PME, and calving year as fixed dependent variables. To describe the association between TW and milk production, a linear mixed model calculated the 305-day milk estimate least square means for the calf outcome variable. This model included farm and DIM as random effects, 305ME was the covariate, while calf outcome, parity group, month and year of calving, gestation length, and calf outcome by parity group interaction were included as fixed dependent variables. With the goal of describing possible associations of TW with dam’s survival and fertility, Cox’s proportional hazard models were calculated. For both survival and conception, censoring happened at 305 days of the lactation following the calving event, and hazard ratios (HR) were calculated for culling and conception. Both models included the following dependent variables: calving month, calving year, farm, gestation length, parity group, calf outcome, previous days open category, previous dry period days, 305PME, and calf outcome interaction by parity group. As early and late cullings might have different implications, the culling model was also calculated, censoring at 60 DIM. The significance level was set at *p* < 0.05; interaction terms were retained in all models if their *p*-value was <0.10.

## 3. Results

### 3.1. Descriptive Results

Descriptive results regarding total number of considered calvings/year, number of herds categorized by number of calvings/year, cow parity groups, and calving outcomes are reported in Table 1.

Among twinnings, 28% of calvings gave birth to females only, 32% to males only, and 40% to males and females.

Reproductive management was based on a combination of estrus detection and ovulation synchronization protocols. Overall, cows were submitted to AI after a mean voluntary waiting period (VWP) of 77 ± 12 days. At the farm level, the shortest VWP was 69 days and the longest 92 days. After the end of the voluntary waiting period, nine farms inseminated cows via estrus detection, while the rest used Double-OvSynch or PreSynch-OvSynch protocols for insemination.

### 3.2. Risk Factors for TW

In the multiple logistic regression model analyzing risk factors for TW, all the variables, except calving year, were significantly associated with TW. Twinning occurrence varied among farms, ranging from 0.2% to 8.9%, with a median of 3.9% (95% C.I 0.35–0.45%), and it was associated with herd factor (*p* < 0.0001) and with 305PME (*p* < 0.05).

Risk factors associated with TW in the present study are reported in Table 2.

### 3.3. Associations Between TW and Milk Production, Fertility, and Survival

Twinning cows showed a shorter gestation length compared to cows calving an SF and an SM (*p* < 0.0001). Twinning was associated with a lower estimated 305-day milk yield (*p* < 0.0001). In the linear regression model, the calving year, herd, calving season, and gestation length variables were all statistically significant (*p* < 0.0001). As the interaction between calf outcome and parity was significant, parity groups were analyzed separately, and the negative association between TW and milk production was confirmed only for LACT = 2 cows; LACT = 2 cows with twinning produced significantly less compared to LACT = 2 cows with singletons and to LACT = 3+ cows with twins or with singletons (*p* < 0.0001). In the LACT = 2 group, also SF calvings showed a lower estimated 305-day milk yield compared to SM calvings (*p* = 0.009). No differences were detected in the 305EP between LACT = 3+ cows with twinning or singletons.

In the present study, twinning was associated with subsequent fertility and survival (*p* < 0.0001). Pregnancy risk for cows calving twins was lower compared to both SF and SM. The odds for culling were increased for cows with TW compared to SF and SM, both in a short-term (60 DIM) and long-term (305 DIM) analysis. Estimated least square means (and 95% C.I.) regarding gestation length and 305-days milk yield production and estimated relative risk (and 95% C.I.) regarding culling risk and pregnancy risk associated with calving outcome are reported in Table 3.

## 4. Discussion

The occurrence of twinning observed in the present study (3.79%) falls within the range reported in the past (and more recently) by many authors [17,18,21,24,25]. The observed twin birth rate could have been artificially increased by the removal of parity 1 cows as parity is directly linked to an increased risk for twinning, as already reported [22,26]. This was confirmed also in the present study since data analyzed considering the lactation group as dependent variable showed that the OR for twinning was 1.6 in LACT = 3+ cows compared to LACT = 2 cows. Nevertheless, LACT = 3+ cows in this study accounted for less than 30% of the total, with 72% of cows being LACT = 2.

Although the twinning rate is not the true reflection of multiple ovulations and vice versa [2], the higher TW rate in cows with higher parity is generally due to a greater double ovulation rate, which increases directly with age (and parity) and milk production. Lopez et al. [27] showed that cows that produced less than 40 kg/day had a very low double ovulation rate, whereas cows producing above 50 kg/day had more than a 50% double ovulation rate. The reason for this phenomenon is related to the fact that as age and parity increase, milk yield grows, and so does the dry matter intake. Cows with a higher dry matter intake have chronically increased hepatic blood flow and metabolism, resulting in lower progesterone (P4) levels [28,29] that increase the likelihood of double ovulation and, therefore, of twinning [30]. The 40% of TW in the present study gave birth to a female and a male calf, being necessarily the results of multiple ovulations, and in the literature, it is reported that >90% of twins in bovine species are non-identical [31]. Another reason that might have contributed to the TW rate was limiting data analysis to the solely Holstein breed. Several studies reported that Holstein Friesian has the highest twinning frequency in dairy cattle, while in contrast, Jersey shows the lowest twinning rate [32].

The range of TW occurrence among herds in the present study was consistent with those reported in the past [3,11], although the extensive adoption of fertility programs and genetic selection for reproductive traits such as daughter pregnancy rate in the dairy industry may have altered the incidence of twin births in recent years. The herd factor was significantly associated with TW risk, and this underlines that TW rate is not a biologically fixed feature but is influenced by many factors specifically linked to reproduction management, cows’ genetic value, and milk production, that greatly vary from herd to herd.

The 305PME was significantly associated with TW risk in the present study, as elsewhere reported [3]. It is anyway clear that the effect of milk production on ovulation is mostly related to the level of production within the 2 weeks before the cow ovulates and not to the total milk production during the entire lactation. In the literature, there is strong physiological evidence that milk yield close to conception is positively associated with the risk of multiple ovulation [27] and, therefore, with TW risk. Specifically, cows that conceive closer to the peak milk may have a greater probability of multiple ovulation and twinning than later in their lactation [21]. From a practical standpoint, these data can be considered as a useful guideline for determining the optimal voluntary waiting period after calving. It a sort of disconnection has been reported between the circulating steroid hormones and the size of the follicles and CL in lactating cows, possibly due to the fact that follicles and CL are less steroidogenically active in lactating dairy cows [33], and the reduction in circulating insulin-like growth factor-1 detected in lactating dairy cows could be related to this reduced steroidogenic capacity [34]. Lopez et al. [27] also reported that the first post-partum ovulation differed from other ovulations, showing a high double ovulation rate that was unrelated to milk production.

In the present study, we found that cows conceiving early in lactation (<100 DIM) had lower TW risk compared to cows conceiving later in lactation, while no difference was found between cows with 100–200 and >200 days open. Consistently, other authors found that double ovulations were less likely to occur in cows showing higher milk production [35] and that cows reaching estrus during the early and late lactation period were 0.56 and 0.84 times less likely to become double ovulators than those showing signs of estrus in the mid-lactation period. It is possible that cows conceiving later (100–200 and >200 days open) already represented a less fertile subpopulation, with an increased rate of ovarian cycle irregularities and double ovulations, and a contributing factor may also be the decreased use of synchronization protocols during this period.

In the logistic regression model examining the relationship between TW and many risk factors, only calving year was not significant, as possibly expected. The analysis of two consecutive years reduces the likelihood of management, environmental, and genetic changes that may affect the data results. Differences in the TW rate between calving seasons were instead detected in the present study and already reported in the literature. The highest twinning rate was observed for cows conceiving in the fall and summer season, and the lowest frequency was observed in the spring and winter season, in agreement with previous studies [11,36]. Many factors, such as feeding management, nutrition level, light period, and temperature, are probably important triggers for the effect of season on TW occurrence. Decreasing daylight hours at the time of conception (negative photoperiod) was found to increase the likelihood of twin pregnancy by being associated with greater multiple ovulation [37,38], while other authors attribute seasonality of TW to feed supplementation during the fall season [35]. Disturbed ovarian activity due to heat stress seems to be another plausible explanation for seasonality in TW rate in cows [37]. However, when comparing results from different studies, it is important to keep in mind that location is significant, as the hours of light and average temperatures vary greatly depending on the geographic area.

Concerning the duration of gestation, as a natural consequence of higher concentrations of cortisol, cows with twins showed a shorter gestation in the present study, which is considered as a risk factor for stillbirth, retained placenta, and, consequently, also for postpartum diseases [1]. Incidence of stillborn or postpartum diseases was not included in the present data analysis, so speculation about the possible negative consequences of shorter gestations in twinning cows is not possible. Twinning has also been reported as a cause for decreased milk production in the subsequent lactation [11,39]; consistently, a lower 305-day milk yield was recorded in the TW cows of the present study (LSM: 11,094 kg) when compared to cows bearing singletons (LSM: 11,306 kg). As the interaction between calf outcome and parity used in the linear regression model was significant, the 305-day milk production was analyzed by parity group separately, and the negative association between TW and milk yield was detected for LACT2 but not for LACT3+ cows. The 305-day LSM recorded in LACT2 cows with twins was the lowest of all groups (10,856 kg) as it was lower compared to milk production in LACT2 and LACT3 cows bearing singletons (LSM: 11,228 kg and 11,384 kg, respectively), but also compared to LACT3+ cows with twins (LSM: 11,332 kg). Therefore, in the present study, the negative association between TW and subsequent lactation performances was evident only for parity 2 cows. This result differs from the data reported by Schambow et al. [21], who found a negative association between twinning and milk production in parity 1 cows (not enrolled in the present study), no association in parity 2 cows, and, surprisingly, a positive association in parity 3+ cows. A possible explanation may lie in the fact that in the statistical model, herd, calving year, and season were also significant; therefore, the “moment” when calving occurred may have weakened or strengthened the association between TW and milk production. It is important to emphasize that differences in milk production between cows with twins versus cows with singletons is worsened by the fact that cows with twins were also at higher risk of culling and postpartum diseases and therefore contributed less milk production data than cows with singletons.

In LACT2 group, cows giving birth to SF also produced less than cows bearing SM. This result is in agreement with studies from France [40] and Denmark [41] but in contrast with other studies [42,43,44]. These latter found that the lactation initiated by the birth of a female rather than a male calf was associated with a higher milk yield; specifically, the female calf gender had carry-over effects associated with higher milk yield in second lactations for Holstein Friesians. They hypothesized that the higher yield in female calves could be due to differences in lactation length, with lower estimates of the whole lactation yield when lactation curves are shifted to the right by 2 days, as happens for male calves. On the contrary, in the present study, cows bearing males produced more. Some authors reported that milk yield is higher when the calf born is heavier [45], and male calves have been shown to be heavier than female calves [46]; this could lead to the appearance of gender-biased milk production when the increased milk yield is due to males tending to be heavier at birth than females. Chew et al. [47] found that calf gender had no significant effect on total milk, fat, or solids when birth weight was included in prediction models, and birth weight had a positive linear relationship with yield traits.

In the present study, twinning was associated with a 28% and a 23% reduced pregnancy risk (censored at 305 days) when compared to SF and SM calvings, respectively. Results from the same dataset describing calving ease showed that pregnancy risk within the first 150 DIM was reduced in cows with assistance at calving compared to those not requiring assistance [48]. A twin calving is often a calving that requires intervention, and this can have negative consequences both on short- and long-term fertility. Previous studies [11,49] also found that fertility was affected by twinning; inseminations per conception were increased for cows that had twins, and the same occurred for incidence of retained fetal membranes, metritis, and abortion [11,17]. Another explanation is that cows who had twins in the previous calving may also present multiple ovulations in the following lactation; although the heritability of TW in cows is low [26], some risk factors (namely, increasing parity and age, low P4, and season) may recur, maintaining a high risk of twinning, which is linked to a higher risk for pregnancy loss. In studies comparing pregnancy loss for twin versus single pregnancies, late embryonic and early fetal losses were greater for cows diagnosed with twins (OR = 2.0–3.7) [50,51], and this may contribute to a reduced pregnancy risk as measured in the present study.

Lastly, an association between TW and culling risk was found. Data censored at 305 days showed that TW cows had a 1.4 OR for culling compared to SM cows and a 1.6 OR compared to SF, consistent with the data from Andreu-Vazquez et al. [2]. Other cow variables significantly associated with culling risk within the first 60 DIM in the present study were herd, parity, season of calving, pregnancy length, previous dry period days, and 305PME. The negative association was even increased when censoring data at 60 DIM; the OR for culling in TW cows increased to 1.6 for TW cows compared to SM, and to 1.9 when compared to SF. At this timepoint, 7.8% and 9.4% of SF and SM cows were culled, respectively; these results align with the general culling rate by 60 DIM, i.e., close to 8%, reported by Dechow and Goodling [52]. Regarding TW cows, the culling rate by 60 DIM in the present study was 17%. This basically means that a TW cow faces almost a double risk of being culled before 60 DIM compared to cows bearing singletons. Pinedo et al. [53] investigated culling reason across breeds and found out that early lactation (<60 DIM) is a critical period mainly for culling due to “died” and “injury-sick” reasons, while specific reasons contributing the most to advanced lactation culling are “low productivity,” “breeding,” and “abortion” (cows were likely diagnosed open during the pregnancy check at dry-off). Interpreting the results from this perspective would indicate that TW affects survival for many reasons, which are different according to the censoring point; in early lactation, TW may increase culling due to calving problems and postpartum disorders, while its long-term negative effect in late lactation could be related to the reduced milk production and fertility.

Considering what has been reported so far regarding the negative consequences of TW on health, well-being, and economic performance in dairy herds, it is essential to pay attention to the development of the genomic prediction for twinning that has been recently implemented [54]. This prediction should be included as a breeding tool for producers in order to improve health, fertility, and production traits in parallel with reducing TW.

In the present study, data were derived from a non-random selection of a limited number of dairy herds, impairing the generalizability of the results. Moreover, being a retrospective analysis, some information—i.e., pregnancy losses, individual heat detection method, or synchronization protocol—is bound to be missing, limiting the power of the study.

## 5. Conclusions

To the best of the authors’ knowledge, the present study is the first to describe twinning on a large dataset in Italian Holstein cattle. Twinning showed high rates in some herds. Parity, season of conception, previous lactation 305-day mature equivalent, and previous calving-to-conception interval were found to be associated risk factors. In agreement with previous studies, results from this study showed that twinning in Italian Holstein breed was associated with a reduced gestation length, negatively affecting milk production and fertility in the following lactation, and consequently, increasing the short- and long-term culling risk. The reduced productive lifespan in Holstein cows, meaning lower total milk yield and increased costs associated with breeding heifers for replacement, may seriously affect the profitability of a dairy herd; therefore, the management and prevention of twinning should be specifically addressed, especially in herds with a high twinning rate.

## Figures and Tables

**Table 1:** Descriptive results of herds enrolled in the study.

Variable	Categories	N (%)
Calvings	2019	20,903 (47%)
	2020	23,632 (53%)
	Total	44,535 (100%)
N calvings/herd/year	<500	28 (45.9%)
	500–100	21 (34.4%)
	>1000	12 (19.7%)
	Total	61 (100%)
Parity	LACT = 2	18,815 (42%)
	LACT = 3+	25,720 (58%)
	Total	44,535 (100%)
Calving outcome	Single female	20,362 (45.72%)
	Single male	22,485 (50.49%)
	Twins	1688 (3.79%)
	Total	44,535 (100%)

**Table 2:** Risk factors associated with TW occurrence in the present study.

Risk Factor	Category	N	TW (%)	Odds Ratio (95% C.I.)	Significance
Parity	LACT = 2	18,815	2.72%	Referrent	*p* < 0.0001
	LACT = 3+	25,720	4.57%	1.66 (1.48–1.85)	
SEACON	Spring	10,745	2.95%	Referrent	*p* < 0.0001
	Summer	8479	4.23%	1.50 (1.28–1.75)	
	Autumn	12,269	4.68%	1.57 (1.36–1.82)	
	Winter	13,042	3.36%	1.1 (0.96–1.29)	
Previous lactation days open (days)	<100	21,859	3.13%	Referrent	*p* < 0.0001
	100–200	17,461	4.34%	1.35 (1.21–1.51)	
	>200	5146	4.72%	1.47 (1.26–1.72)	

SEACON: season of conception.

**Table 3:** Gestation length, estimated 305-day milk production, culling risk, and pregnancy risk by calving outcome.

Variable	Singleton Female	Singleton Male	Twins	Significance
Gestation length (days): LSM (95% C.I.)	276.5 ^a^ (276.4–276.6)	277.8 ^a^ (277.8–277.9)	272.2 ^b^ (271.9–272.4)	*p* < 0.0001
305-ME (Kg): LSM (95% C.I.)	11,295 ^a^ (11,127–11,463)	11,318 ^a^ (11,150–11,486)	11,093 ^b^ (10,905–11,281)	*p* < 0.0001
In LACT = 2 cows: LSM (95% C.I.)	11,196 ^a^ (11,027–11,366)	11,259 ^b^ (11,089–11,429)	10,855 ^c^ (10,637–11,073)	*p* < 0.001
In LACT = 3+ cows: LSM (95% C.I.)	11,394 ^a^ 11,225–11,564	11,376 ^a^ 11,207–11,548	11,333 ^a^ 11,139–11,525	
Culling risk RR in the first 60 DIM (95% C.I.)	Referrent	1.18 (1.00–1.26)	1.90 (1.66–2.17)	*p* < 0.0001
RR in the first 305 DIM (95% C.I.)	Referrent	1.11 (1.06–1.16)	1.57 (1.43–1.73)	*p* < 0.0001
Pregnancy risk by 305 DIM RR (95% C.I.)	Referrent	0.94 (0.91–0.97)	0.72 (0.66–0.78)	*p* < 0.0001

LSM: least square means; 305-ME: estimated 305-days milk production; LACT = 2 cows: parity 2 cows; LACT = 3+ cows: parity ≥3 cows; RR: relative risk; DIM: days in milk; ^a, b, c^: different letters within the same row correspond to significant differences.

## Data Availability

The data that support the findings of this study are available from the corresponding author upon reasonable request.
